# SBRT in the Very Elderly: A Viable Option for Pulmonary Oligometastases?

**DOI:** 10.3390/cancers17152512

**Published:** 2025-07-30

**Authors:** Samuel M. Vorbach, Meinhard Nevinny-Stickel, Ute Ganswindt, Thomas Seppi

**Affiliations:** Department of Radiation Oncology, Medical University of Innsbruck, 6020 Innsbruck, Austria

**Keywords:** pulmonary oligometastases, stereotactic body radiotherapy, very elderly, radiotherapy

## Abstract

Background/Objectives: The global population of individuals aged ≥ 80 years is rapidly growing, leading to an increasing incidence of cancer diagnoses in this age group. While stereotactic body radiotherapy (SBRT) has proven effective in treating pulmonary oligometastases, patients over 80 remain underrepresented in clinical analyses. This study aimed to evaluate clinical outcomes and toxicity of SBRT for pulmonary oligometastases in octogenarians. Methods: This retrospective, single-centre analysis included 34 patients aged ≥ 80 years treated with SBRT for histologically confirmed pulmonary oligometastases between 2010 and 2024. Results: A total of 46 pulmonary metastases were treated with curative intent using fractionation schemes of 3 × 15 Gy, 6 × 8 Gy, or 10 × 6 Gy. Median biologically effective dose (BED10) was 112.5 Gy. Follow-up included regular CT imaging and toxicity assessment according to CTCAE. With a median follow-up of 22.6 months, 1-, 2-, and 3-year local control (LC) rates were 95.2%, 95.2%, and 90.2%, respectively. Median overall survival (OS) was 46.6 months, with 1-, 2-, and 3-year OS rates of 78.4%, 71.4%, and 59.5%. Progression-free survival (PFS) at 1, 2, and 3 years was 63.4%, 51.6%, and 47.3%, respectively. No grade ≥ 3 toxicities were observed. Grade 2 pneumonitis and dermatitis occurred in 2.9% each and were well managed. Asymptomatic rib fractures were detected in 5.9% of patients. No significant predictors for LC, PFS, or OS were identified in univariate analysis. Conclusions: SBRT for pulmonary oligometastases in patients ≥ 80 years is feasible, safe, and effective. High local control, favourable cancer-specific survival, and minimal toxicity support its use as a curative-intent treatment in this growing patient population. These findings contribute important site- and age-specific evidence and support the inclusion of very elderly patients in future prospective SBRT trials.

## Simple Summary

The population of individuals aged ≥ 80 years is rapidly increasing, leading to more cancer diagnoses in this group. Stereotactic body radiotherapy (SBRT) is an established treatment for pulmonary oligometastases, but data for the very elderly are limited. We retrospectively analysed 34 patients aged ≥ 80 years (median 83) with 46 histologically confirmed pulmonary oligometastases treated with SBRT (median BED10: 112.5 Gy). Median follow-up was 22.6 months. One-, two-, and three-year local control rates were 95.2%, 95.2%, and 90.2%. Median overall survival was 46.6 months; 3-year cancer-specific survival was 70.9%. No grade ≥ 3 toxicities occurred; grade 2 pneumonitis and dermatitis were rare (2.9% each) and manageable. No significant predictors for local control or survival were identified. SBRT is safe, effective, and well-tolerated in patients aged ≥ 80 years with pulmonary oligometastases, supporting its use as a curative-intent option in this growing demographic.

## 1. Introduction

The global population is ageing rapidly, with individuals aged 80 years and over representing an increasingly important demographic group: it is estimated that there will be 265 million people aged 80 years and over by the mid-2030s [1], and the global population aged 80 years and older is expected to triple by 2050 [2]. This demographic shift has profound implications for healthcare systems worldwide, particularly for cancer care, as age remains the most important risk factor for developing cancer [3,4].

The number of cancer patients in the very elderly demographic group is increasing rapidly. In 2022, an estimated 2.6 million new cancer cases across all disease stages (14% of all diagnoses) and 2.1 million cancer deaths (22% of all cancer deaths) occurred in adults aged 80 and older [2]. This proportion is expected to increase dramatically, with estimates suggesting that by 2050, about 6.9 million new cancers will be diagnosed in adults aged 80 years and over, accounting for 20.5% of all cancer cases [5].

Despite the growing burden of cancer in the very elderly patients, this population remains severely underrepresented in the clinical trials that drive treatment guidelines. For example, while people aged 70 and older make up about 42% of the overall cancer population, they represent only about 24% of the participants in clinical trials submitted to the FDA, and less than 10% of patients aged 70 years and older are enrolled in National Cancer Institute trials [6]. Another analysis of 302 randomised clinical trials found that across all oncological diseases, trial participants were significantly younger than the general patient population, with a median age difference of 6.49 years [7]. Even when included, these elderly trial participants tend to be fitter than the average older patient, with fewer comorbidities or impairments [8]. This underrepresentation means that the evidence that guides modern cancer treatment is largely based on younger, healthier populations and may not be fully generalisable to frail older patients. There is a clear need for studies focused on the very elderly to provide the best possible treatment decisions for this growing cohort.

Several recent studies have aimed to better characterise the barriers contributing to the exclusion of older adults from cancer research and treatment. Ludmir et al. [9] analysed 742 oncological randomised controlled trials comprising 449,720 participants and found that 10.1% included an upper age limit, with a median cut-off of 72 years (interquartile range 70–80), thereby explicitly restricting access for older patients. Tang et al. [10] conducted a population-based study of 677 individuals aged ≥ 65 years with metastatic lung cancer (median age 84) and found that 53.4% would typically be excluded from trials, most commonly due to poor performance status, cardiac disease, or a history of prior malignancy. Patel et al. [11] conducted a comprehensive analysis of modern phase III oncological randomised controlled trials to evaluate the incidence and factors associated with exclusion criteria related to prior malignancy within the past five years (PMEC-5). PMEC-5 criteria were identified in 41% of trials, occurring more frequently in industry-sponsored studies and those assessing targeted therapies. The presence of PMEC-5 was independently associated with increased age disparities. Their findings suggest that PMEC-5 criteria likely contribute significantly to age-related disparities in cancer trial enrolment.

Beyond trial design, older patients also face disparities in real-world treatment. In a systematic review of 102 observational studies, Bastiaannet and Pilleron [12] found that adults aged ≥ 60, particularly those over 80, were significantly less likely to receive standard therapies such as surgery, chemotherapy, and radiotherapy, irrespective of cancer type or care setting. However, when treated, fit older adults experienced outcomes comparable to younger patients, suggesting they can benefit from standard therapies when appropriately assessed. These considerations are equally relevant in the management of metastatic disease in older adults, where the evidence supporting local therapies remains scarce.

The lung is one of the most common sites of metastatic disease in cancer patients. Autopsy studies have shown that up to half of patients who die from extrathoracic malignancies have pulmonary metastases (PMs) [13,14]. Common primary tumours that metastasise to the lung include breast, colorectal, renal, head and neck cancer, and sarcomas [14,15]. Specifically, the diagnosis of oligometastatic disease is becoming more common, largely driven by advances in imaging technologies and more effective systemic therapies that extend the survival of cancer patients [16]. The term ‘oligometastatic disease’ was first introduced by Hellman and Weichselbaum [17] in 1995, and refers to an intermediate state between localised cancer and advanced disease. It is currently most commonly characterised by the presence of five or fewer metastatic lesions that are amenable to targeted local therapies. The clinical rationale for treating such lesions with local therapies is based on the hypothesis that managing oligometastatic disease can delay progression, prolong survival, and possibly lead to long-term remission in carefully selected patients [18]. Among these approaches, stereotactic body radiotherapy (SBRT), also known as stereotactic ablative body radiotherapy (SABR), has become a widely used, non-invasive treatment option. SBRT achieves excellent local control rates of over 90% for lung metastases and is generally well tolerated, with grade 3 or higher toxicities reported in approximately 5% of patients in several studies [19,20].

The potential for curative treatment in selected patients with oligometastatic disease has been strongly supported by the landmark SABR-COMET trial [21], a randomised, multicentre phase II trial that compared standard palliative treatment with SBRT to all known metastatic sites in patients with a controlled primary tumour and 1–5 metastases. SBRT significantly improved overall survival (OS), with median survival increasing from 28 to 41 months, and a significant proportion of patients remained progression-free at long-term follow-up [22].

However, it is important to recognise that the SABR-COMET trial, like many pivotal oncology trials, included relatively few very elderly patients. The oldest patient enrolled was 75 years old. Therefore, the applicability of these trial findings to patients aged 80 years and older remains somewhat uncertain. In real-world clinical practice, this very elderly population is often discussed in multidisciplinary tumour boards (MDTs) and often deemed ineligible for systemic therapy or pulmonary metastasectomy due to frailty, comorbidities, or poor performance status [23]. In these cases, SBRT may be a uniquely suitable alternative due to its non-invasive nature and limited treatment burden. However, robust data on the outcomes and toxicity of SBRT specifically in octogenarians with pulmonary oligometastases remain limited. Two retrospective studies included patients aged 65 years and older [24] and 70 years and older [25], respectively. While one study has focused exclusively on patients aged 80 years and older [26], all three studies have one common limitation: the inclusion of patients with metastatic lesions to multiple anatomical sites, which limits the site-specific interpretation of toxicity and outcome data for lung SBRT.

The aim of the present study was to report the outcomes and toxicities of SBRT for lung oligometastases in patients aged over 80 years.

## 2. Materials and Methods

### 2.1. Study Population

Between January 2010 and January 2024, 34 patients aged 80 years or older with histologically confirmed pulmonary oligometastases were treated with SBRT at the Department of Radiation Oncology, Medical University of Innsbruck, Austria. Oligometastatic disease was defined as the presence of up to five lesions suitable for local treatment, and was classified according to ESTRO and EORTC consensus [27].

All cases were reviewed by an institutional multidisciplinary tumour board. All patients had undergone curative-intent standard-of-care treatment of their primary tumour prior to developing lung metastases. Following the detection of lung metastases, CT-guided or bronchoscopic biopsy was routinely performed for histological verification of lung lesions. Patients were subsequently reassessed by the tumour board based on histological findings and assigned to SBRT. All pulmonary metastases were treated with curative intent.

Data were collected on the type of primary tumour, initial treatment, systemic therapy received before or after SBRT, and the time from diagnosis of the primary tumour to the appearance of pulmonary metastases. Demographic data were also collected before an SBRT course. The Charlson Comorbidity Index [28] was calculated to more precisely assess the burden of comorbidities. A SBRT course was defined as all SBRT treatments given within one month.

The study was approved by the ethics committee of the institutional review board of the Medical University of Innsbruck (EC No. approval: 1343/2024). All procedures conducted in this study involving human participants are in accordance with the ethical standards of the institutional review board, as well as with the Helsinki Declaration (1964) and its later amendments or equivalent ethical standards.

### 2.2. Techniques of Radiotherapy

Patients were immobilised in the supine position using the Elekta BodyFIX system. A four-dimensional CT scan was performed to assess tumour location and respiratory motion over time. For target volume delineation, the gross tumour volume (GTV) was defined as the radiologically evident disease and contoured, incorporating co-registered imaging when available. An internal target volume (ITV) was defined to account for respiratory motion effects on the GTV. The planning target volume (PTV) was generated by uniformly expanding the ITV by 5 mm in all directions. Organs at risk (such as the lungs, spinal cord, trachea, bronchial tree, oesophagus, chest wall, and blood vessels) were delineated to minimise radiation exposure. Tumours were classified as central [29] or ultra-central [30] as previously described. Dose prescriptions varied according to the location of the lesion, and the proximity of the lesion to critical structures, with the following regimens applied: 60 Gy in 10 fractions, 48 Gy in 6 fractions, or 45 Gy in 3 fractions. The corresponding dose prescription modalities included the 65% isodose, the 80% isodose, or the 100% isodose. Treatment planning was performed using precisePLAN (Elekta AB, Stockholm, Sweden) until 2013, and subsequently with Pinnacle Software (most recent version V14; Philips Medical, Fitchburg, MA, USA) until the end of the study. SBRT plans were generated to maximise target volume coverage while minimising dose to surrounding organs at risk. Coverage ranged from 0.88 to 1.00 (mean 0.98, 95% CI: 0.97 to 0.99). A conformity index, defined as the ratio of the absolute volume enclosed by the prescribed isodose to the absolute volume of the PTV, was allowed up to 1.6, with a median value of 1.39 and a range from 1.12 to 1.60. Patients were treated with three-dimensional conformal radiotherapy (3D-CRT) or VMAT using an Elekta Synergy linear accelerator until 2013, and a Versa HD linear accelerator thereafter (both from Elekta AB, Stockholm, Sweden). Daily cone beam CT scans were used to verify and, if necessary, adjust patient positioning to ensure metastasis coverage within the PTV.

### 2.3. Follow-Up

After completion of SBRT, follow-up radiological imaging (usually CT scan, PET-CT if necessary) was performed every 3 months for 1.5 years and then every 6 months thereafter. Any equivocal findings were evaluated by the multidisciplinary tumour board and, if necessary, a short-term follow-up including CT scan was initiated. Tumour response was classified according to the Response Evaluation Criteria in Solid Tumours (RECIST) [31], and in the case of metabolic imaging, according to the PET Response Evaluation Criteria in Solid Tumours (PERCIST) [32].

Local control was defined as the absence of progression in the treated lesion and measured as the time from the end of SBRT to either progression or the last imaging follow-up. PFS was defined as the time from the end of SBRT to disease progression, either within or outside the treatment, death from any cause, or the last imaging. For patients with sequential oligometastatic disease who underwent an additional SBRT course, a second PFS was defined as the interval from the end of the second SBRT course to progression. Systemic treatment-free survival was defined as the time from the end of SBRT to initiation of systemic treatment or to death from any cause. OS was measured from the completion of SBRT to death from any cause or the date of the last follow-up. Cancer-specific survival (CSS) was defined as the time from completion of SBRT to death attributable to cancer or the date of the last follow-up. Follow-up time was calculated from the date of completion of SBRT to the date of the last available CT scan. Toxicity was assessed by medical history, physical examination, laboratory tests, and medical imaging and graded according to the Common Terminology Criteria for Adverse Events (CTCAE).

### 2.4. Statistical Analysis

Descriptive analysis was used to summarise relevant patient and treatment characteristics. LC, PFS, systemic treatment-free survival, CSS, and OS were assessed using the Kaplan–Meier method. Univariate and multivariate analyses of potential prognostic factors for clinical outcomes were performed using the Cox proportional hazards model. Multivariate analysis was performed by using the rule of stepwise backward elimination of non-significant factors. *p* values < 0.05 were considered to be significant. The linear quadratic model was used to calculate the respective biologically effective doses (BEDs) for all radiotherapy prescription doses, assuming an alpha/beta ratio of 10 (BED10). All statistical analyses were performed using SPSS Statistics (V26, IBM Cooperation, Armonk, NY, USA).

## 3. Results

### 3.1. Patient Population

This study included 34 patients with a total of 46 pulmonary oligometastases treated with SBRT. The median follow-up time was 22.6 months (range, 2.6–86.1 months). The median time from diagnosis of pulmonary oligometastases to SBRT was 36 days (range, 8–78 days).

Patient demographics, characteristics of lung metastases, and treatment details are summarised in Table 1. The median age at the start of SBRT was 83 years (range, 80–92 years), and the majority of patients were male (70.6%). Most patients had an ECOG performance score of 0–1 (84.2%) and a Charlson Comorbidity Index of 0–2 (76.3%).

Primary tumours were predominantly colorectal (41.2%), followed by head and neck (11.8%), non-small cell lung cancer (8.8%), gynaecological (8.8%), and various other types (29.4%). The median interval between diagnosis of the primary tumour and diagnosis of lung metastases was 27.5 months (range, 1–245 months). Most patients (80.6%) had received no systemic therapy prior to SBRT. At the time of SBRT, no patients received concomitant systemic therapy.

Of the 46 metastases treated, 31 (67.4%) were present as single metastases at the time of SBRT, while the remaining 15 metastases (32.6%) were treated simultaneously in groups of 2 or 3. Lesions had a median diameter of 13 mm (range, 6–42 mm) with common locations in the right lower lobe (26.1%) and right upper lobe (23.9%). Most metastases were peripheral (80.4%), with few central (15.2%) or ultra-central (4.3%) lesions.

The median PTV was 16.0 cm^3^ (range, 4.5–97.8 cm^3^). Dose prescriptions included 3 × 15 Gy (n = 28, 60.9%), 10 × 6 Gy (n = 5, 10.9%) and 6 × 8 Gy (n = 13, 28.3%), resulting in a median biologically BED10 at the PTV periphery of 112.5 Gy (range, 86.4–112.5 Gy).

### 3.2. Outcomes

**Figure 1:**
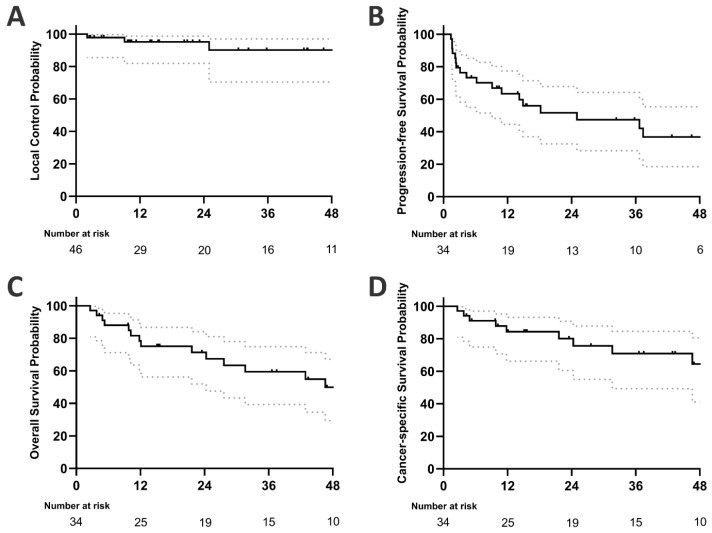
Kaplan–Meier plots showing (**A**) local control (46 treated pulmonary metastases), (**B**) progression-free survival, (**C**) overall survival, and (**D**) cancer-specific survival of 34 patients aged ≥ 80 years (dotted grey lines indicate 95% confidence interval).

Among all patients who underwent SBRT, the LC rate at one, two, and three years was 95.2% (95% CI: 82.0 to 98.8%), 95.2% (82.0 to 98.8%), and 90.2% (70.4 to 97.0%), respectively (see Figure 1). On univariate analysis, none of the factors examined—primary tumour type, BED10, or the status of oligometastatic disease—were identified as significant predictors of local control in SBRT-treated pulmonary oligometastases (see Table 2).

One-, two-, and three-year progression-free survival rates were 63.4% (44.5 to 77.4%), 51.6% (32.4 to 67.8%), and 47.3% (28.3 to 64.1%), respectively. Univariate analysis did not identify any of the factors studied as significant predictors of progression-free survival (Table 3). Four patients (11.8%) developed sequential oligometastatic disease following SBRT treatment of their initial pulmonary oligometastases. Each of these patients subsequently received a second course of SBRT, resulting in a second median progression-free survival (PFS) of 8.5 months (range 2.5–54.9 months). A total of seven patients (20.6%) received systemic therapy after SBRT due to further disease progression. The median time to initiation of systemic treatment was 9.4 months (range 2.9–78.1). Systemic treatment-free survival was 62.3% (43.2 to 76.6%) at one year, 62.3% (43.2 to 76.6%) at two years, and 46.9% (30.6 to 67.5%) at three years.

Median OS was 46.6 months. OS at one, two, and three years was 78.4% (59.9 to 89.1%), 71.4% (52.0 to 84.1%), and 59.5% (39.3 to 74.9%), respectively. Univariate analysis did not identify any significant predictors of overall survival (Table 3). A total of 21 patients died during follow-up, of whom 13 had progressive tumour disease. The remaining eight patients died of causes unrelated to their oligometastatic cancer: three died of cardiovascular disease, three of infections, and two of unknown causes but with no evidence of tumour progression before death. Accordingly, cancer-specific survival was 84.3% (66.2% to 93.2%) at one year, 80.1% (60.5% to 90.7%) at two years, and 70.9% (49.3% to 84.6%) at three years.

### 3.3. Toxicities

SBRT was well tolerated in this clinically fragile cohort, with no grade 3 or higher toxicities observed. All patients completed the treatment without interruption. One patient (2.9%) developed grade 2 pneumonitis, which was effectively treated with corticosteroids; the patient subsequently returned to baseline activity levels and reported no increase in his pre-existing dyspnoea. One patient (2.9%) developed grade 2 radiation dermatitis, which was adequately controlled with topical therapy.

During follow-up, three patients (8.8%) reported a subjective increase in pre-existing dyspnoea, all of whom had underlying chronic obstructive pulmonary disease. Conversely, two patients (5.9%) with baseline dyspnoea experienced symptomatic improvement after SBRT. Asymptomatic rib fractures were detected in two patients (5.9%) on routine follow-up CT imaging, both of which were already consolidated at the time of diagnosis, and neither patient reported pain or required clinical management. No patients reported persistent or severe fatigue during the follow-up.

## 4. Discussion

This retrospective single-centre analysis offers valuable real-world evidence on the role of stereotactic body radiotherapy in a highly specific and understudied population: patients aged 80 years and older with histologically confirmed pulmonary oligometastases. Our findings demonstrate excellent 3-year local control (90.2%), favourable cancer-specific survival (70.9%), and a remarkably low toxicity profile, with no observed grade ≥ 2 adverse events. These results provide strong support for the safety, efficacy, and feasibility of SBRT in a very elderly cohort traditionally underrepresented in clinical trials.

Our outcomes align with and extend previous research across different studies. In evidence, Scorsetti et al. [25] reported 1- and 2-year LC rates of 86.8% and 76.3% in elderly patients with mixed metastatic sites (median age 79), alongside a 2-year OS of 72.0% and minimal toxicity. Compared to our study, which focuses exclusively on lung metastases and includes an older cohort (median age 83), we observed similarly favourable survival and an even more favourable safety profile. This difference may be partially attributable to the anatomical focus and consistently high BED10 values in our protocol (median 112.5 Gy).

The report of Cuccia et al. [26] represents one of the few studies to exclusively examine SBRT in octogenarians; however, only 30% of their patients have the lung as the treatment site. With 61 patients aged ≥ 80, they reported LC rates of 98.8% and 88.2% at 1 and 2 years and no grade ≥ 2 toxicity. Our results echo this efficacy and tolerability, although with slightly lower 1-year LC (95.2%) but longer median OS (46.6 months vs. 20 months). Our exclusive focus on pulmonary lesions and uniform multidisciplinary assessment may have contributed to more durable outcomes.

Lancia et al. [24] examined SBRT in elderly patients with isolated metastases (median age 75) and highlighted the feasibility of short-course “blitzkrieg” regimens, showing 2- and 3-year LC of 87% with manageable toxicity. Our study confirms that even older cohorts can benefit from these stereotactic strategies.

Landmark studies such as SABR-COMET [22] have established SBRT as an effective strategy in selected oligometastatic patients, but have largely excluded those aged ≥ 80. In contrast, our work bridges this gap by offering site-specific, age-specific data, complementing broader trials while addressing their limitations in geriatric representation. On the other hand, our data is supported by the SABR-COMET trial, also demonstrating that a substantial number of patients can actually be treated curatively with SBRT, because they show progression-freedom still after years post-SBRT of the lung.

Together, these comparisons highlight several important topics. First, across all known studies, chronological age alone does not preclude patients from benefiting from ablative local therapy since there is growing evidence of prolonged PFS and OS at moderate toxicity risk. Second, the toxicity profile of SBRT remains favourable even in the oldest-old, supporting its consideration over more invasive or systemic options. Third, while data from mixed anatomical sites are informative, our study’s focus on pulmonary lesions enables a clearer interpretation of lung-specific side effects—key for clinical decision-making in thoracic oncology.

Importantly, our study also adds nuance to the discussion around treatment sequencing. We observed that 20.6% of patients eventually required systemic therapy, but with a median delay of 9.4 months. This underlines the potential of SBRT to defer systemic treatment, preserving quality of life and limiting toxicity—an aspect of particular relevance in elderly care.

Nonetheless, limitations remain. The retrospective nature and small sample size introduce possible selection biases, and our findings, while compelling, would benefit from validation in prospective trials incorporating geriatric assessment tools and quality-of-life metrics. In addition, the median follow-up in our cohort may be insufficient to fully capture all late toxicities. However, given the advanced age and limited life expectancy of this patient cohort, long-term follow-up is inherently constrained.

## 5. Conclusions

Our study demonstrates that SBRT is a safe, effective, and durable treatment option for pulmonary oligometastases in patients aged ≥ 80 years. SBRT offers a curative-intent modality in a population often considered ineligible for systemic therapy. These findings, placed alongside a growing body of literature, strongly advocate for the inclusion of octogenarians in future SBRT trials and for a paradigm shift toward individualised local therapies in geriatric oncology.

## Figures and Tables

**Table 1:** Patient, lesion, and treatment characteristics.

Characteristic		Value or No (%)
**Total no. of patients**		34
**Total no. of metastases**		46
**Sex**	Male	24 (70.6%)
	Female	10 (29.4%)
**Age at start of SBRT**	Median	83
	Range	80–92
**ECOG performance status at SBRT**	0	12 (31.6%)
	1	20 (52.6%)
	2	6 (15.8%)
**CCI at SBRT**	0–2	29 (76.3%)
	3–5	9 (23.7)
**Primary tumour**	Colorectal	14 (41.2%)
	Head and neck	4 (11.8%)
	NSCLC	3 (8.8%)
	Gynaecological	3 (8.8%)
	Other	10 (29.4%)
**Time between diagnosis of the primary tumour and diagnosis of PM (months)**	Median	27.5
	Range	1–245
**Systemic therapy prior to SBRT**	Yes	7 (19.4%)
	No	29 (80.6%)
**Oligometastatic classification at the time of PM diagnosis**	Synchronous oligometastatic	6 (15.8%)
	Metachronous oligorecurrence	25 (65.8%)
	Repeat oligorecurrence	7 (18.4%)
**SBRT courses per patient**	1 course	30 (88.2%)
	2 courses	4 (11.8%)
**PMs treated per SBRT course**	Total number of patients (PMs)	38 (=46 PMs; 100%)
	Patients with 1 PM per SBRT course	31 (=31 PMs; 67.4%)
	Patients with 2 PMs per SBRT course	6 (=12 PMs; 26.1%)
	Patients with 3 PMs per SBRT course	1 (=3 PMs; 6.5%)
**Diameter PM (mm)**	Median	13
	Range	6–42
**PM Location**	Right upper lobe	11 (23.9%)
	Right middle lobe	3 (6.5%)
	Right lower lobe	12 (26.1%)
	Left upper lobe	9 (19.6%)
	Lingula	2 (4.3%)
	Left lower lobe	9 (19.6%)
**PM Location**	Peripheral	37 (80.4%)
	Central	7 (15.2%)
	Ultra-central	2 (4.3%)
**PTV (cm^3^)**	Median	16.0
	Range	4.5–97.8
**Single dose prescribed**	Median	15
**(PTV encompassing, Gy)**	Range	6–15
**Total dose prescribed**	Median	45
**(PTV encompassing, Gy)**	Range	45–60
**BED10 at PTV periphery (Gy)**	Median	112.5
	Range	86.4–112.5

SBRT: stereotactic body radiotherapy; ECOG: Eastern Cooperative Oncology Group; CCI: Charlson Comorbidity Index; PM: pulmonary metastasis; PTV: planning target volume; BED: biologically effective dose.

**Table 2:** Univariate analysis of factors related to local control.

Factor	HR (CI 95%)	*p* Value
**Primary tumour**		
CRC	1 (reference)	
Other	0.52 (0.05–5.94)	0.602
**BED10**		
<100	1 (reference)	
>100	0.28 (0.03–3.12)	0.302
**PTV size in ml**		
≤16	1 (reference)	
>16	0.54 (0.05–6.04)	0.620

**Table 3:** Univariate analysis of factors related to PFS and OS.

	Univariate Hazard Ratio for PFS		Univariate Hazard Ratio for OS	
Factor	HR (CI 95%)	*p* Value	HR (CI95%)	*p* Value
**Sex**				
Male	1 (reference)		1 (reference)	
Female	1.27 (0.48–3.35)	0.636	1.38 (0.55–3.46)	0.491
**CCI**				
0	1 (reference)		1 (reference)	
≥1	0.99 (0.40–2.45)	0.987	0.80 (0.32–1.96)	0.618
**Primary tumour**				
CRC	1 (reference)		1 (reference)	
Other	1.24 (0.50–3.12)	0.639	0.88 (0.37–2.11)	0.771
**Number of PM**				
1	1 (reference)		1 (reference)	
>1	1.56 (0.58–4.23)	0.379	1.19 (0.46–3.12)	0.721
**Systemic therapy prior to SBRT**				
No	1 (reference)		1 (reference)	
Yes	1.90 (0.71–5.07)	0.198	0.77 (0.25–2.31)	0.638

## Data Availability

The data presented in this study are available upon reasonable request from the corresponding author.
